# Safe Circular Food Systems: A Transdisciplinary Approach to Identify Emergent Risks in Food Waste Nutrient Cycling

**DOI:** 10.3390/foods13152374

**Published:** 2024-07-27

**Authors:** Brieanne Berry, Travis Blackmer, Michael Haedicke, Susanne Lee, Jean D. MacRae, T. Reed Miller, Balunkeswar Nayak, Louis Rivet-Préfontaine, Deborah Saber, Linda Silka, Astha Thakali, Jared Wildwistle, Chyanne Yoder, Cindy Isenhour

**Affiliations:** 1 Environment & Sustainability, Ursinus College, 601 E Main St, Pfahler Hall, Collegeville, PA 19426, USA; bberry@ursinus.edu; 2 School of Economics, University of Maine, 5782 Winslow Hall, Orono, ME 04469, USA; travis.blackmer@maine.edu; 3 Sociology, University of Maine, 5728 Fernald Hall, Orono, ME 04469, USA; michael.haedicke@maine.edu (M.H.); louis.rivetprefrontaine@maine.edu (L.R.-P.); 4 Senator George J. Mitchell Center for Sustainability Solutions, University of Maine, 5710 Norman Smith Hall, Orono, ME 04469, USA; susanne.lee@maine.edu (S.L.); silka@maine.edu (L.S.); 5 Civil and Environmental Engineering, University of Maine, 5571 Boardman Hall, Orono, ME 04469, USA; jean.macrae@maine.edu (J.D.M.); reed.miller@maine.edu (T.R.M.); astha.thakali@maine.edu (A.T.); 6 Food Science and Human Nutrition, School of Food and Agriculture, University of Maine, 5763 Rogers Hall, Orono, ME 04469, USA; balunkeswar.nayak@maine.edu; 7 School of Nursing, Florida Southern College, 111 Lake Hollingsworth Dr., Lakeland, FL 33801, USA; deborah.saber@baycare.org; 8 Gulf of Maine Research Institute, 350 Commercial St, Portland, ME 04101, USA; jwildwistle@gmri.org; 9 Anthropology & Environmental Policy, University of Maine, 5773 South Stevens Hall, Orono, ME 04469, USA; chyanne.yoder@maine.edu; 10 Anthropology and Climate Change Institute, University of Maine, 5773 South Stevens Hall, Orono, ME 04469, USA

**Keywords:** food waste, nutrient cycling, compost, digestion, risk, trust, safety, transdisciplinary, systems thinking

## Abstract

With growing awareness of the environmental, economic, and social costs associated with food waste, there is a concerted effort on multiple scales to recover the nutrient value of discarded food. These developments are positive, but the rapid movement toward alternatives and the complexity of solving problems located at the intersection of economic, social, and environmental systems also have the potential to produce unanticipated risks. This paper draws upon long-term stakeholder-engaged research throughout New England, with a focus on Maine, to develop a transdisciplinary, systems-based model of the potential social, economic, and environmental risks of food waste nutrient cycling. Our effort is intended to help inform the creation of safe, functional, and environmentally benign circular food systems.

## 1. Introduction

In recent years, there has been a surge of interest in food loss and waste in the United States, with particular attention to its economic, environmental, and social costs. (According to the UN’s World Food Program, “food loss” refers to foods that are damaged or destroyed in the supply chain prior to distribution to the consumer. “Food waste” refers to foods that are discarded by retailers, food service providers, and consumers. In our conceptual model, we are concerned with all sources of food that make their way into nutrient cycling processes, including both food loss and waste. We utilize food waste to refer to both. Most estimates suggest that each year approximately 30% to 40% of all the food produced for human consumption in the US is lost or wasted [1,2,3]. Annual food wastage rates in the US exceed those in most other countries, including countries at a similar stage of economic development [4]. Agriculture in the US is governed by a problematic paradox: “agricultural productivity and competition through trade keeps prices low, making waste economically rational for many consumers” [5]. However, this comes at great *economic cost, nationally.* Americans spend USD 218 billion each year to plant, grow, process, transport, and then dispose of food that is never eaten [2].

Not only are wasted nutrients no longer available for human consumption, but the resources embodied in that food (money, fuel, water, and human labor) are also lost and result in significant *environmental costs*. Researchers have estimated that the amount of energy associated with these losses is equivalent to the “annual petroleum available from drilling the outer continental shelf” [6]. Further, organic materials decompose anaerobically in landfills, producing methane, which constitutes 16.4% of all U.S. methane emissions [7], and creating leachate that can contaminate groundwater [8].

Finally, there are significant *social costs* associated with food waste. We produce an abundance of food in the United States, more than 50 million tons each year, yet 11% of the population is food-insecure, including more than 6 million children [9]. Scientists have estimated that 25 million Americans could be fed each year by recovering and redistributing just 15% of food losses annually [10]. Under current agroeconomic conditions, the problem of food security is exacerbated as significant proportions of nutrients are lost through food waste. With a rapidly growing global population, sustainable agriculture depends on food waste reduction and nutrient recovery [11]. Landfilled food may also increase the exposure of nearby communities to noxious odors, waste traffic particulates, or groundwater contamination. These burdens disproportionately affect minority communities [12].

The states of New England have set some of the most ambitious goals for reducing food waste in the US [13], in the interest of moving toward more circular food systems that eliminate waste and are regenerative by design [14]. States in the region have undertaken environmental education efforts, implemented food waste recycling laws, established food waste reduction targets, and made significant investments in food redistribution programs, composting, and digestion technologies [15,16,17,18,19,20].

In this paper, we draw upon a decade of community-engaged research with a diverse network of stakeholders (e.g., food waste producers, waste haulers, landfill operators, composters, digesters, hunger relief organizations, environmental groups, regulators) throughout New England to focus specifically on efforts to cycle food waste nutrients back into agricultural soils. Since beginning our work in 2014, we have learned that sometimes solutions with the best intentions can present new risks. This research was therefore driven by a primary research question: what are the social, economic, and environmental risks associated with expanding food waste nutrient cycling in New England?

Our findings make it clear that systems-based and transdisciplinary approaches (that include the expertise of the people working in the system) are necessary to ensure that risks are not simply displaced from one part of the system to another or from one group of actors to another. Clean, nutrient-rich soils, free of contaminants and toxins, are essential for safe agricultural production and public health. Access to safe and clean soil becomes a matter of justice [21,22]. It is imperative that any food waste-derived agricultural supplements are carefully managed to ensure safety, trust, and fair availability.

This paper makes two primary contributions toward this goal. First, our systems-based approach synthesizes stakeholder perspectives and the existing interdisciplinary academic research to identify potential risks associated with transitions toward stronger food waste nutrient cycling systems. While there is a significant amount of scientific research on the environmental and ecological implications of food waste nutrient cycling, there is very little research that incorporates the social sciences to gather stakeholder perspectives and even fewer that also incorporate consideration of the social and economic dimensions of nutrient cycling. Stakeholders in various positions frequently have contrasting perspectives and levels of concern about potential risks, complicating decision-making and necessitating tradeoffs between social, economic, and environmental priorities. It is important to consider the perspectives of these experts who are likely to determine the ultimate success of efforts to expand food waste nutrient cycling. Levels of risk also depend heavily on a whole array of mediating factors. In order to address the subjective forms of risk experienced by people throughout the food system, our systems-based approach examines the structure as well as the interactions, flows, and feedback between—and amongst—the various stages (and people) in food waste nutrient cycling operations in New England [23]. As Kibler and colleagues argued in their review of food waste management alternatives, “Characterizing the complex problem of postdisposal…impacts of wasted food, including descriptions of dynamic feedback behaviors, presents a significant research gap and a priority for future research” [24]. Our second contribution is, then, to conceptually model the potential risks associated with food waste nutrient recycling systems specifically in Maine where the landfilling and incineration of food waste is currently allowed but legislation has been proposed to require food waste nutrient cycling. We examine risks produced through and at the intersections of (1) the food waste generation source; (2) sorting; (3) collection methods; and (4) processing technologies. We argue that this systems-based and stakeholder-engaged approach is important to collaboratively anticipate, prevent, and plan for a full range of potential risks. These risks must be identified and mitigated to ensure that rapidly developing circular food systems are safe, fair, and sustainable.

## 2. Background, Materials, and Methods

### 2.1. Food Waste Nutrient Recycling and Policy Momentum in New England

**Figure 1:**
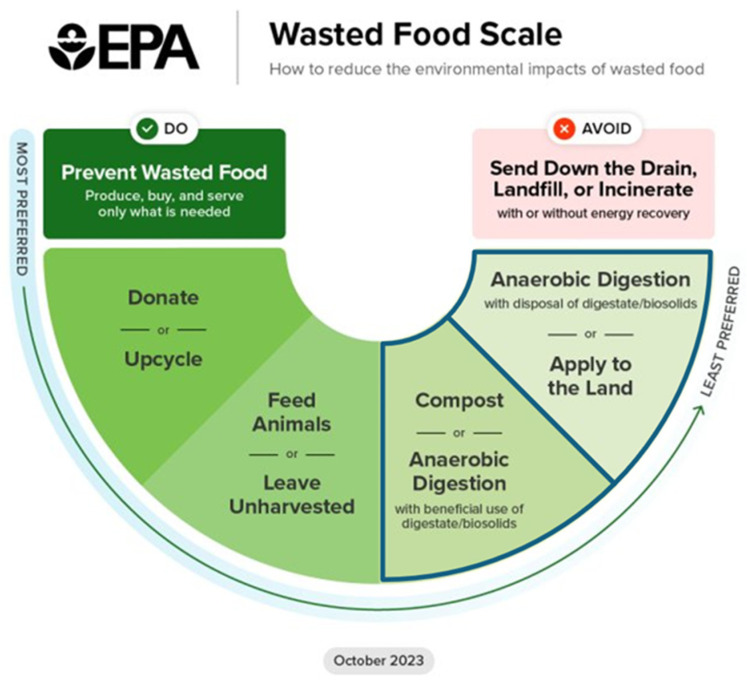
EPA Wasted Food Scale. Our focus areas are outlined.

The Environmental Protection Agency’s (EPA) Wasted Food Scale [25] provides an organizing structure meant to guide food loss and waste reduction efforts. It is designed to maximize positive social, economic, and environmental outcomes (Figure 1). The scale prioritizes food waste source reduction, followed by recovery for human consumption (donate or upcycle), recovery for animal consumption (feed animals or leave unharvested), and recycling (compost or anaerobic digestion) before landfilling or incineration.

In this paper, we focus on two segments of that scale—food waste nutrient cycling processes, including digestion and composting. Both of these processes produce agricultural supplements. Our focus on nutrient recycling is motivated by recent policy momentum generated by state and local governments. While the Wasted Food Scale clearly encourages source reduction and redistribution ahead of food waste recycling, strategies further to the right side of the scale (Figure 1) are prioritized in practice [26]. Policies to promote food waste reduction and recovery through composting and anaerobic digestion have been considered at the national level [27], but policy uptake and implementation is moving particularly quickly at municipal and state levels. Cities across the United States have implemented food waste recycling policies, largely focused on curbside food scrap collection and drop-off programs [28]. In the United States, the number of households with access to organics collection via curbside pickup, drop-off locations, or both grew from 2.7 million in 2013–2014 to 14.9 million in 2023 [29]. Developments at the state level have rapidly shifted materials management systems by expanding infrastructural capacity for nutrient cycling [30]. Since 2011, several New England states—Massachusetts, Vermont, Connecticut, Rhode Island, and New Hampshire—have enacted policies to ban food waste from landfills [31].

The investment of resources toward food waste recycling, rather than redistribution, is related to a number of factors including the maturity of these technologies [32], the logistical complications stemming from the perishable nature of food [33], potential business donors’ fear of liability [34], and the propensity to favor more convenient and less costly options [35]. These barriers clearly need to be addressed to support the preferred solutions on the EPA Wasted Food Scale. Composting and anaerobic digestion, while not as beneficial as waste reduction or food recovery efforts, extract residual nutrient value from food while seeming to skirt difficult questions related to safety, making them attractive for risk-averse institutions looking for more immediate, inexpensive, and convenient means to divert waste.

The rapid expansion of food waste recycling capacity is frequently advanced without much attention to the potential risks, well documented in the existing literature [36]. For example, biological contaminants can present a threat to human and animal wellbeing [37]. While the USDA and US Compost Council have developed comprehensive voluntary testing protocols for toxic components such as heavy metals and pathogens [38], most states do not require extensive testing for composters or digesters, many of which make their products available for agricultural applications. Moreover, testing for the many different contaminants that can pose potential risks is highly cost-prohibitive. Further, the costs of recycling processes and materials relative to virgin nutrients can create market-based risk [39,40]; and the redistribution of nutrients can affect soil quality and biodiversity through land degradation, erosion, and acidification [11]. It is also possible that technocentric solutions can be counterproductive, actually increasing demand for “water, energy, and inputs” [41].

The bulk of studies addressing nutrient recycling risks utilize a technological and material approach [42,43,44] that neglects the numerous social agents within food production systems—from farmers, consumers, and corporations to state agents [39]. But processes of production, packaging, and consumption that constitute food systems are entangled in a complex network of ecological, economic, and social actors [41,44] that must also be considered.

The material transformation of food waste into valuable nutrients via processes of digestion and composting requires parallel conceptual transformations [45]. New England’s food waste recycling system consists of both a material food waste hierarchy and social value regimes [46], necessitating an integrated analytical approach. This paper’s transdisciplinary framework thus synthesizes stakeholder perspectives and practices with a systems-based structural focus to conceptualize nutrient recovery risks as an assemblage of economic, environmental, and social processes [44,45].

These various risks merit attention, not to forestall innovation or progress for food waste nutrient cycling, but rather to proceed carefully and with intention. Food waste has been described as a “wicked problem” given complex connections to overlapping economic, social, and environmental systems and because solutions that address one aspect of the problem can have unintended consequences in another [47]. Further, various actors within systems often develop competing solutions, based on the prioritization of *contrasting social, economic, or environmental goals* [26,48].

### 2.2. Materials and Methods

Research focused on socio-ecological systems makes it abundantly clear that the management of isolated aspects of fundamentally interconnected systems can result in unintended systemic consequences [24]. Indeed, complex problems—like food waste—are difficult to solve [47] because they can produce “emergent risks” that arise “from the interaction of phenomena in a complex system” [49]. Given the need for solutions that work at the complex intersection of agriculture, environmental health, food insecurity, waste management, environmental protection, and food economics, the University of Maine Materials Management Research Group was established in 2014 to work with community partners on the creation of more sustainable materials management systems, including the reduction of food waste and diversion of organic materials from landfills.

Our team—composed of scholars and students in economics, ecology and environmental sciences, social psychology, environmental engineering, food science, business, nursing, sociology, and anthropology—has since built a wide network of over 400 stakeholders throughout New England through a series of workshops, surveys, and interviews. This group includes town and municipal managers, landfill operators, waste haulers, composters, digesters, state regulators and legislators, regional planners, hunger relief agencies, and a whole array of food waste producers, retailers, distributors, and large institutions like colleges, hospitals, and schools. Our collective work, with many of these partners, has established a wide range of programs to address food waste, ranging from cooking demonstrations to community sharing programs.

In this paper, we draw on a subset of our work to focus specifically on the potential risks associated with the rapid movement toward food waste recycling. Our methods include observations and insights generated through a series of six stakeholder workshops focused on sustainable materials management; a working meeting of key stakeholders engaged in a wide range of food waste reduction efforts; a survey of state-certified food waste recycling facilities in New England; and follow-up interviews with managers at composting and digestion facilities.

In 2014 and 2015, we organized a series of six workshops drawing together representatives from state government, municipalities, and the waste management industry (*N* = 130). Large group and small group discussions were captured by note-takers and synthesized into workshop reports that were made available for all participants to review and amend. Participants were asked to envision a more sustainable materials management system and quickly honed in on the importance of addressing food waste [36]. In August of 2017, we convened a workshop focused specifically on food waste reduction with stakeholders in Maine (*N* = 32), one of the few states in the New England region which, at the time of publication, had not required food waste recycling. Participants represented a broad range of interests and perspectives in food waste nutrient cycling systems. While the aim of that meeting was to discuss and evaluate policies designed to reduce food waste more generally, stakeholders drew upon their knowledge of neighboring state programs to identify several barriers, tradeoffs, and risks associated with emerging food waste recovery and nutrient cycling systems in New England [50].

We also developed a survey, specifically focused on observed and perceived risks of contamination associated with food waste generated by a wide variety of actors—as well as strategies for mitigation. This survey was disseminated via email to all composting and digestion facilities licensed to accept food waste in Massachusetts, Vermont, and Maine. According to publicly available lists obtained directly from state environmental protection agencies in MA, VT, and ME, there are 114 facilities licensed to receive food waste in these three New England states. We received 32 responses to our survey, for a 28% response rate [32]. Data were analyzed using descriptive statistics in Excel, given the small sample size.

Finally, we reached out to all survey participants to request a follow-up interview. Six facilities volunteered to participate in a 30 to 60 min interview focused on observations and perceptions of potential risks as well as current and potential mitigation strategies [51]. All qualitative data including interview transcripts and workshop notes were analyzed using manual inductive thematic coding.

## 3. Results: Potential Risks

In the sections below, we draw on both existing research and our stakeholders’ perspectives to outline three forms of potential risks—economic, environmental, and social—associated with the movement toward stronger food waste recycling systems (Table 1).

### 3.1. Economic Risks

Workshop participants in Maine were more likely to mention the potential economic risks associated with food waste nutrient cycling than concerns about environmental or social risks. Historic dependence on landfilling and incineration has created a “lock-in” effect that can make investments in a more circular food system seem economically risky, particularly given how cheap it is to landfill [52]. However, the historically higher landfilling costs in New England compared to the Midwest/Central/Southern parts of the United States [53] may contribute to the drive to divert more food waste in the region.

#### 3.1.1. Infrastructure Investments and Economic Incentives

Different stakeholder groups have different economic incentives that can make investments in food waste recycling more or less risky. Waste generators often associate these shifts with higher taxes and fees to dispose of food wastes. Waste managers anticipate extra expenses associated with building the infrastructure necessary to collect, sort, and process an additional waste stream, since the overwhelming majority of food waste is still incinerated or landfilled. Many waste managers spoke about the tradeoffs between the higher value of clean, separated materials necessary for operationalizing more circular systems and the costs associated with separate food waste collection and processing mechanisms for food wastes.

The scalable flexibility of composting requires a smaller initial investment compared to digestion technologies, but both depend on having adequate feedstock and markets for residuals. One processor described the economic risks that new composting facilities face:

“…*it’s actually a tougher business to get into than you think. You can do it with a bucket […] on a tractor, but if you’re trying to make really high-end compost you need a turner and you need a screen and those are two very expensive pieces of equipment. **And you can’t buy equipment like that and then only compost 1000 yards a year. It does not work like that. You have to be moving some volume to support that equipment** […] Some people do a good job with a bucket, but just to give you an idea, a small trommel screen—used—is in excess of $200,000. **If you want to make really good compost and have that as the output you’ve got to be serious about it and you have to have real steady flow on the inbound side and the process to make it all equal on the outbound side and a good sale price***”.

*(Interview, 2018 [emphasis ours])*

Smaller municipalities that centralize collection and pay by the ton have a distinct set of tradeoffs. There is a potential for immediate cost savings if the cost of diverting food scraps is less than the costs to haul and landfill food waste with the rest of the trash. The fees are easy to assess when an entire truck that arrives at a disposal facility is weighed and that cost is directly attributed to the municipality. Larger municipalities are more likely to be in a long-term contract that offers curbside collection to their residents. In these cases, the collection of an additional waste stream adds costs rarely offset by reduced landfill tipping fees (which are currently the cheapest option). Further, some private waste processing facilities require municipalities to sign contracts guaranteeing the tonnage that will arrive at the landfill, essentially preventing municipalities from adopting more aggressive food waste recycling campaigns for fear of violating “flow control” requirements [54].

Waste from businesses and institutions, such as restaurants or hospitals, is typically collected with waste from other customers on a route designed for efficiency. Hauling and disposal contracts are privately negotiated and typically depend on the size of the dumpster and frequency of collection, not the mass of waste generated. Thus, a restaurant opting to divert food scraps may not obtain any immediate cost savings, nor are they guaranteed to be able to negotiate a lower hauling fee over time.

Many different types of stakeholders note that, without mandatory food waste recycling, there is insufficient incentive to participate since landfill tipping fees are currently the cheapest option for food waste disposal. Many of the policies for mandatory diversion are on the West Coast and in New England where disposal costs are relatively high [53].

#### 3.1.2. Market Risks: Aligning Supply and Demand

**Figure 2:**
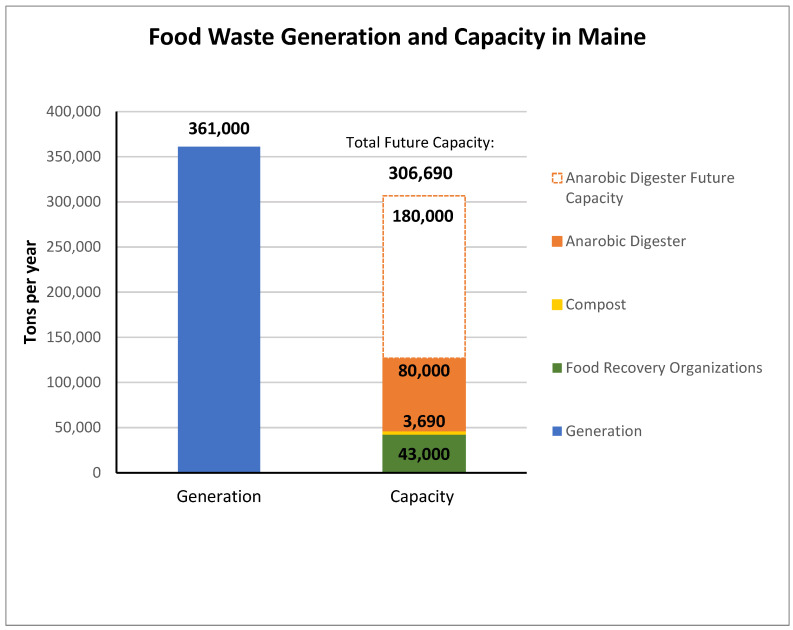
Maine annual food loss and waste generation and recovery capacity.

Beyond concerns about the costs and risks associated with investing in food waste recycling infrastructure, many stakeholders were concerned about market risks, specifically the possibility of a mismatch between supply and demand. Figure 2 illustrates the approximate current annual generation of food loss and waste in Maine, which exceeds recovery capacity. The current and future recovery capacity is dominated by anaerobic digestion; additional capacity is planned beyond the 180,000 tons depicted. Digestion is followed by food banks, with relatively limited composting [55].

Some waste managers were concerned, for example, that waste generators would not participate and thus there would not be adequate feed stock to fuel investments in composting and digestion technologies. Some of our stakeholders noted that existing disposal contracts for municipalities ensure a predictable flow of waste and revenue to a facility, but these contractual entanglements can prevent towns and individuals from separating food scraps from other waste [50]. Without adequate incentives and policy support, private and public investments in food waste processing technologies are perceived as too risky. These concerns are less of an issue in states with stronger policy guidance (e.g., MA, VT) that guarantee a stock of inputs than in states without clear policy guidance (e.g., ME).

Other stakeholders identified the risk of bottlenecks in operations if adequate capacity does not exist at one phase in the process or in a particular geography. They argued that enacting policy to support or require food waste recycling *without* sufficient infrastructure can create bottlenecks and system failures if food waste inputs overtake infrastructural capacity [50]. Composting and digestion capacity are important, but so are other processes including transportation, storage, and pre-processing technologies. One stakeholder noted that when Massachusetts implemented their commercial food waste landfill ban, they provided incentives for compost and AD technologies, but no incentives for pre-processing (e.g., grinding, de-packaging). This oversight led to an initial bottleneck that could have been avoided with greater understanding of existing capacity. The economic risks at play create a thorny double-bind, where uncertainty about potential participation makes it difficult to invest in food waste processing facilities, while uncertainty about processing capacity relative to waste generation and participation makes it difficult to create transformative policies that assure sufficient participation. Finally, some expressed concern about the risk that there may not be an adequate market for finished products. Many of these risks vary depending on the business model adopted for collection (drop-off, subscription pick up, municipal pick up) and end use (compost sales, community-utilized, farm-utilized).

Pilot food waste collection programs in Maine have observed fluctuating participation rates, ranging from a low mean weekly set-out rate of 29.5% to a high mean weekly set-out rate of 43.7% [50]. Being able to anticipate the volume of food waste inputs is important for planning and investing in food waste recycling. The EPA estimates that approximately 15% of the US waste stream is food scraps [53]. However, many waste generators may not be willing or able to participate. Farrell and Jones [56] suggest that individuals may choose not to participate in food recycling programs because they lack trust in the system and have overall negative attitudes concerning waste management. Participation is also likely to vary geographically, making it hard to estimate the volume of food waste to be processed. Yet, if processors concerned about adequate volume take more risky materials like slurry from de-packaged foods or post-consumer food waste streams, they also run risks associated with the contamination of feedstocks, both in terms of physical (visible) contaminants and trace contaminants. Critically, these risks interact with each other. Focusing on increasing infrastructural capacity, then, is not likely to improve overall outcomes in food waste diversion without attention to the other risk factors that may impact the decisions of processors.

### 3.2. Social Risks

In order to ensure the success of nutrient cycling systems for food waste, public participation and support are essential. Waste generators must follow separation protocols to reduce contamination, while potential customers, gardeners, and farmers must trust that the nutrients recovered from food waste are safe and healthy to use for food production. Communities need to trust that nuisances and odors will be managed and that programs will be implemented in just and fair ways that minimize social risks in host communities. Indeed, the project of developing a more circular food system through the land application of food waste nutrients is “incomplete unless the earning of public trust in the practices is included” [57]. Without the support of the local community, facilities can face issues with odor, traffic, and nuisance complaints. One interviewee discussed this risk explicitly, describing a company that had invested hundreds of thousands of dollars in a facility only to shut down when “*the neighbors went cuckoo*” (Interview, 2018). Indeed, without significant community engagement and support, many facilities around the country have been forced to close [58,59,60]. Engaging with the public and earning trust must not occur “as an afterthought”, but instead in the early stages of research, planning, and implementation [57]. Research on a wide array of sustainability programming has demonstrated how, without parallel attention to social sustainability, programming can reproduce inequalities and even exacerbate unsustainable practices [61]. Stakeholders participating in our research rarely mentioned social risks but those who did expressed two concerns, detailed below.

#### 3.2.1. Subverting the Food Waste Scale, Undermining Hunger Relief and Waste Reduction

First, several stakeholders expressed concern that, without alternative incentive structures, large investments in nutrient cycling might divert attention away from food waste reduction efforts or discourage the redistribution of still edible foods to those experiencing hunger [26]. This effect may be heightened by the tendency among institutions like universities, schools, or municipalities to “keep quiet” about food insecurity since they have limited means to address the problem [62]. In contrast, composting and other food recycling strategies are useful for establishing a green identity or brand. When coupled with concerns about liability issues, food waste generators may prioritize recycling over redistribution, which can exacerbate existing food insecurity. Once contracts are signed, generators are often held responsible for delivering certain quantities of feedstock, potentially creating lock-in effects and encouraging food waste generators like grocers, restaurants, and large institutions to generate food waste for nutrient cycling rather than redistribute still edible foods. A strong focus on nutrient cycling might also distract attention from important conversations about preventing waste in the first place—essentially subverting the Wasted Food Scale and avoiding some of the most important and systemic drivers of food waste.

Research suggests that “pro-environmental behaviors” (like composting) can have, in some circumstances, unintended negative effects (like diverting attention away from more substantial changes) [63,64]. For example, if a household is confident that they are taking great strides to compost their waste, they may be less inclined to worry about reducing food waste through more sustainable shopping (negative spillover). Likewise, if a grocer is recycling food waste, they may be less inclined to invest time and effort to donate to hunger relief organizations. Conversely, if a food waste generator becomes more aware of the need to reduce waste as a result of composting and begins more careful food procurement planning (positive spillover), these effects can have environmental, economic, and social benefits. While there are few studies of food waste recycling spillover effects, one study found no significant spillover from composting behavior to food waste prevention behaviors, positive or negative [65]. The empirical documentation of the effects of Vermont’s universal recycling law suggests that the requirement for waste generators to divert food waste from landfills resulted in an unanticipated 40% growth in donations to hunger relief organizations, with significant positive social benefits [66]. More recently, research participants representing hunger relief organizations in Maine told us that many of the donations they are receiving are marginally or not edible. While food generators can receive tax benefits for donating unsellable foods, all too often hunger relief organizations cannot distribute the food and these organizations with small budgets are forced to bear the cost of disposal.

#### 3.2.2. Unequal Distribution of the Environmental Costs and Benefits of Food Waste Recycling

Secondly, some stakeholders expressed a concern that public investments in nutrient cycling could be unfair particularly if they are large and centralized. As surplus food becomes increasingly commoditized, there are risks that smaller players may be squeezed out of opportunities to obtain and process wasted food [26]. Others identified the risk that rural municipalities would not receive as many benefits given that they would have to pay considerable sums to haul food waste for processing or invest in their own infrastructure while larger, more populated urban areas are more likely to benefit from centralized public investments due to population density and transportation efficiencies. Research suggests that there is a higher potential for urban waste operations to be sited in low-income or minority neighborhoods or to contribute to their devaluation after siting [67,68], potentially leading to social, economic, and environmental justice issues like increased traffic, pollution, and nuisance odors. Despite these risks, several studies have recently found that distributed community composting programs, if designed carefully and with intention, can be both financially feasible and result in other positive social and community benefits [69,70].

### 3.3. Environmental Risks

Research related to the risks associated with food waste recycling for agricultural applications is rapidly emerging [71,72]. Our own work has demonstrated contamination from PFAS and antibiotic resistance genes in food waste destined for field application [73,74]. As Gillett wrote nearly 30 years ago, “The more MSW composting is accepted as a waste disposal option (in contrast to the somewhat more limited production of a useful soil amendment), the more serious becomes the issue of whether total risk has been broadened excessively” [75].

Our workshops and interviews with stakeholders suggest that research participants overwhelmingly viewed the environmental dimensions of food waste recycling in a positive light—as a drastic improvement compared to the environmental impacts of landfilling food waste [51]. However, this disjuncture between stakeholders’ perceived risks and levels of contamination reported by scientific research merits attention. Here, we outline potential environmental risks and contaminants.

#### 3.3.1. Physical Contaminants

Food waste processors receiving municipal food scraps are particularly concerned about the risks of “the big three”—plastic, glass, and produce stickers. Produce stickers create a very strong reaction among food waste recyclers. Because they do not break down in the composting process they act as “colorful contamination flags in the finished compost” [76]. Plastics may be ground finely in soil amendments but become increasingly visible as the soil is “washed” by precipitation, raising concerns about contamination and eroding trust in food waste-derived soil amendments.

Results from our survey of food waste processors support these findings (*N*= 32). In an open-ended question about the contamination risks associated with accepting food waste, trash—including plastics, straws, utensils, and fruit stickers—was identified most frequently. Trash presents economic risks to processors, whose outputs may be difficult to market if they are visibly contaminated. Some respondents also noted the high cost of screening contaminants from finished compost, particularly straws and fruit stickers. Processors also mentioned more than economic risks associated with plastic contaminants. One interviewee discussed the potential environmental and health risks associated with plastic contaminants:

“*There is a certain type of plastic I haven’t nailed it down, it’s either number 5 or like number 3 or something that tends to fragment in the compost piles and gets to be these really small flecks of plastic. If it was micro leaching or something, that would be a problem. And I don’t know about that*”.

*(Interview, 2018)*

This response highlights how materials received by processors are often unknown. Some processors actively reject loads with plastic contaminants to avoid loss of value for their products [77]. The increased use of “de-packaging” machines could lead to an increased level of microplastic contamination. Though identified as extremely difficult to measure, researchers have begun to tackle this topic. While recent studies have shown that nearly 50% of samples had some form of microplastic contamination, the varying methods of detection and measurement do not allow for these outcomes to help inform policy [78].

Similarly, glass fragments present a significant liability. Even one shard of glass “ruins the batch” (Stakeholder Survey, 2018), and can lay waste to an entire season of work. When end-products contain shards of glass, they become not only economically risky but processors noted that broken glass presents health risks to their employees and to end-users, while other materials, like steel wool, can kill livestock. Survey respondents commented that there is “no solution” for decontaminating materials once glass has been introduced (Stakeholder Survey, 2018).

Some physical contaminants are riskier for digesters, which require predictable and consistent feedstocks to protect their process and equipment. Our survey of processors suggests that while composters are more concerned about contaminants that are visible in the end-product, digesters tend to be more concerned with physical contaminants that can damage equipment such as stringy or fibrous waste that can bind machinery or grease and grit that can block pumps and nozzles.

#### 3.3.2. Trace Contaminants

The existing literature also suggests that a number of non-visible contaminants can present significant economic, environmental, and social risks to food waste processors and end-users. These contaminants include pathogens, heavy metals, pesticides, and anaerobic digestion process inhibitors, like ammonia or salt.

Pathogens and antibiotic-resistant microbes pose health and economic risks to food waste processors, although these risks were mentioned less frequently than physical contaminants by survey participants. One processor commented on the uncertainty surrounding pathogens, writing that “you cannot see them, we do not test for them and they likely present the highest liability issue however remote that might be” (Stakeholder Survey, 2018).

The literature suggests a range of human health risks associated with pathogens. While a United Kingdom study found no increased risk of disease for individuals living closer to composting facilities compared to those living further away [79], pathogens can affect those who come into direct contact with the food waste as it decomposes. Microbes, such as *Legionella* (a bacterial pathogen), have been found to cause pathology after exposure to composted material [80]. There are approximately 50 species of *Legionella*, but infections are most commonly caused by *L. pneumophila*, which causes pulmonary infections [81]. However, other strains are found in composted material produced under increased temperature and humidity conditions [82]. *Legionella longbeachae* has caused cellulitis in immunocompromised patients after exposure to composted potting soil [83]. Another microbe that is present in the high-temperature phase of the compost cycle, and is an opportunistic pathogen of humans, is *Aspergillus fumigatus* [84]. One interviewee noted that composting regulators see the high-heat processes of composting and digestion as “their ultimate end-all, be-all for, I’ll call it bad stuff, say, like GMOs or pathogens or whatever” (Interview, 2018), yet while composting may kill many microbes, other micro-contaminants can persist.

Unmetabolized antibiotic substances in food and pharmaceutical products may also end up in soil and water, and finally in the food chain [85]. The presence of antibiotics has been reported in municipal sewage, in the effluent of sewage treatment plants, and in surface water [86,87] as well as in food waste samples. The development of antibiotic-resistant bacteria in soil is one of the greatest concerns with regards to the residential and institutional use and overuse of antimicrobials.

Other micro-contaminants, more likely to be identified as risks by digesters than composters, were process inhibitors such as ammonia, sodium, and sulfur (Stakeholder Survey, 2018). Positive conditions for bacterial assemblages must be maintained during anaerobic digestion, and high levels of salt, ammonia, and acidity can disrupt the digestion process, resulting in the need for time-consuming and expensive fixes to the process. While anaerobic digestion (AD) technology and expertise is mature, when feedstocks vary drastically, maintaining equilibrium within digestion systems is an ongoing challenge and a marked economic risk.

Heavy metals and minerals also present risks to food waste nutrient cycling. The growth and development of crops may also be altered by the input of water-soluble salts in agricultural soil due to the application of food waste-derived soil supplements, much of which is high in salt content (e.g., canned soups, processed foods). Plants fertilized with compost produced with high-salt foods may be unable to acquire water and nutrients from the treated soil due to its high salinity and osmotic pressure [88].

There is also a risk that compost- and digestate-produced fertilizers could be contaminated with heavy metals. Metals persist through food waste recycling processes, so repeated application to agricultural land may result in the uptake of metals into crops. Heavy metals have high reactivity, are toxic to biological systems, and can harm plants and animals [89]. Human health impacts of heavy metal ingestion include bladder, lung, and skin cancer; kidney, liver, and bone impairment; and neurotoxicity [90]. While metals may be immobile in finished compost or digestate, the repeated application of residuals may result in soil or crop contamination [56]. Once contaminants are introduced into the environment, plants can take them up through foliar deposition [91]. While heavy metal transport is dependent on plant species, absorption, retention, plant morphology, and physiology [92], leafy vegetables, in particular, have higher bioaccumulation factors [93,94]. Sometimes, contamination can make its way far into food systems. Gillett [75] argues that we must think beyond the direct application of residuals to the soil, and instead consider the bioaccumulation of metals throughout the food web.

Survey respondents also identified toxins as primary contamination risks associated with accepting food waste. These included pesticides, herbicides, and other toxins that are particularly difficult to detect. One interviewee commented on the challenges of detecting herbicide contamination:

“*you have to specifically ask and pay for each herbicide …, and there are infinite numbers so the best way that I have found to protect us other than controlling your raw materials—where I can go and chase somebody down—is to do bioassays or grow outs*”.

*(Interview, 2018)*

Indeed, several processors we interviewed detailed the tests they order and pay for to identify *individual* contaminants, yet “the diversity and range in scale of such chemicals in municipal solid waste (MSW) practically precludes traditional approaches from establishing a level of safety for exposure to the *total* load” [75]. The United States Composting Council acknowledges the burdens of testing for these contaminants, counseling composters to voluntarily test and report on their feedstock contamination:

“*Composters can test for contamination, but the tests are time intensive if done in-house and expensive if hired to a laboratory. Guarding against contamination requires a great deal of new data collection and record keeping. The USCC believes that it is unfair to place this financial burden on the composter. Compost producers can help us make this argument by testing your feedstocks and products. If you find contamination, you should report it to your state agency AND to the USCC*”.

*[95]*

This is particularly the case with the recent discovery of per- and polyflouroalkyl substances (PFAS) in finished digestate and compost [96]. Associated with coatings that repel oil and water, as found in many food packaging materials, PFAS can be introduced into these processes through biosolids, industrial residuals, and food scraps containing packaging with PFAS. Unfortunately, these mobile, persistent, and bioaccumulative substances are now pervasive in the environment—but there is no known way for processors to remove them from their products. The only way to avoid them is to refuse to accept feedstocks that contain PFAS [73,97] or to lobby the federal government and chemical producers to phase them out [51,98].

Some interviewees lamented, regarding chemicals and pesticides, that “there’s no regulations for it, so no one gives a hoot” (Interview, 2018). For some processors, the risks of chemicals and herbicides extend beyond the legal, economic, and environmental to the moral and personal. An interviewee described their anguish when a farmer unknowingly contributed glyphosate-laced hay to a composting facility:

“…*the poor farmer—he doesn’t know. He has no idea, and then he takes this load of manure and takes it to the composter thinking that he’s doing something great, small farmer, and it’s laced with glyphosate, it’s laced with whatever persistent herbicides…. and you can’t tell unless you do bioassays on [the] finished product*”.

*(Interview, 2018)*

Processors are faced with difficult decisions in the context of micro-contaminants. The economic costs of testing are significant, and further, the literature suggests that individual tests are insufficient. Yet, the economic, environmental, and social costs of producing a soil amendment that can cause human, animal, and environmental harm are also significant. Processors are well aware that their business depends on public acceptance and trust, and they are also aware that invisible contaminants present a real threat that goes far beyond loss of market share or public trust.

### 3.4. A Conceptual Model of Contamination Risk in Food Waste Nutrient Cycling

**Figure 3:**
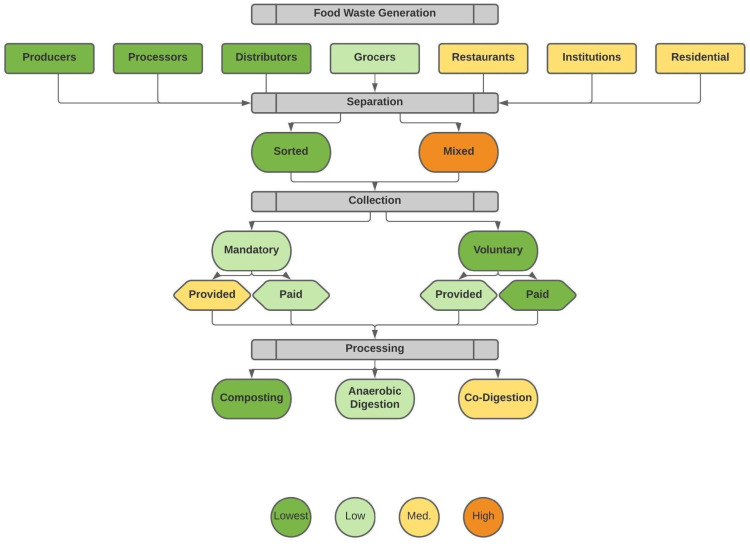
Potential for contamination risks as identified by stakeholders and literature review.

There is potential for social, environmental, and economic risks to emerge in all stages of food waste recovery and nutrient cycling. These risks are highly variable depending on local conditions and a wide range of factors [57,75]. In this section, we draw upon the potential risks outlined in the previous sections to organize a conceptual model of potential risks according to the different stages of food waste recovery and recycling: (A) generation; (B) separation; (C) collection; and (D) processing. Food waste is heterogenous and can travel on many different pathways within a circular food system. While it is useful to map out potential risks according to the flow of materials, it should also be noted that some risks emerge in multiple stages, and others exist in the spaces in between and in interactions and feedback. Further, risks may accumulate over time through repeated cycles. What follows is necessarily a simplification, in an attempt to model and make these emergent risks more explicit (Figure 3).

#### 3.4.1. Risks Associated with Food Waste Generator Type and Waste Profile

Food wastes come from a wide variety of generators. From non-salable produce and expired grocery packages to household leftovers—the generators of food waste are as diverse as their waste streams. The nature, volume, and location of the waste these entities produce can affect the likelihood of contamination. Food waste generated by growers on the farm, for example, tends to be fairly homogenous, typically composed of the non-salable vegetables or fruits they grow, and is often composted on-site, reducing the risk of contamination since growers are likely aware of potential contaminants associated with their inputs or processes. Processors, distributors, and retailers have a more varied waste stream, but a typical load is likely to be fairly consistent. In contrast, restaurants, catering firms, and institutions like schools, colleges, and hospitals produce highly variable waste streams with both pre- and post-consumer wastes and are prone to contamination with a variety of other materials from packaging to plastic cutlery. These large institutional generators and restaurants have a very different food waste profile compared to households. As one food waste processor told us during an interview,

“*I think you have to differentiate. Individuals are residential, that pay for it, they have a tendency to have much lower rates (of contamination). Commercially, in restaurants… I would say even though they’re paying for it, we get a lot of garbage. They’re like trash, forks, knives, you know, plastic bottles. That’s because you’ve got restaurant workers in the back versus a resident at home—and they (residents) want to keep that silverware*”.

*(Interview, 2018)*

**Figure 4:**
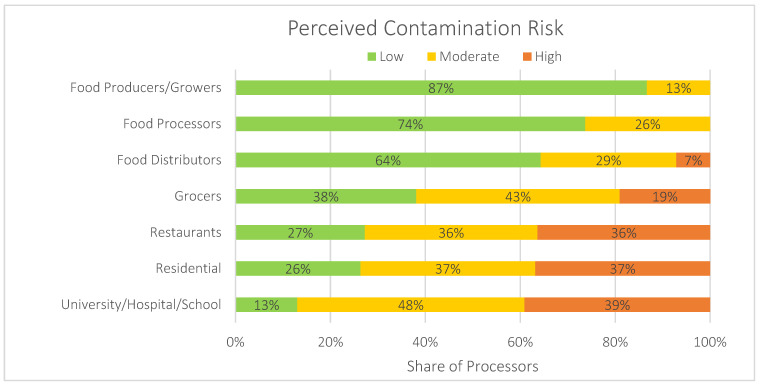
Processor perceptions of level of contamination risk by generator type. (Due to rounding, some figures may not total to 100).

In general, our stakeholders suggest that generators with more diverse waste streams introduce a greater chance of physical contamination (see Figure 4). This relationship, however, is mediated by a number of other factors we outline in the following section, including the extent to which waste streams are kept separate, the extent to which the policy setting demands recovery, and the characteristics of specific organizations and households. One interviewee told us, for example,

“*High schools are awful, elementary schools aren’t much better. Hospitals, I think are all right. I guess it would depend on how much is pre-consumer and how much is post-consumer*”.

*(Interview, 2018)*

While data on contamination rates are scarce for institutions, a Minnesota study found organics with visible contamination in K-12 schools that ranged from 2% to over 20%, with plastic film and beverage cartons representing the most common types of contaminants measured [99]. Another study conducted by a member of our team found that 82% of samples contained non-food waste, some as high as 39% of the sample by mass [100]. Other institutional generators like hospitals have unique properties that result from potential microbial contamination [101], where the contaminants in food could also include the potential for antimicrobial-resistant bacteria (e.g., methicillin-resistant *Staphylococcus aureus* [MRSA], Carbapenem-resistant *Enterobacteriaceae*) [102]. Most hospital waste, which includes surplus food from patients with infectious disease, is classified as unregulated, which may be disposed of in incinerators or landfills, or recycled through composting or anaerobic digestion [103,104,105]. Thakali’s study [74] tested 10 samples of hospital food wastes ready for collection by recyclers, and detected *Listeria monocytogenes* in 2 out of 10 hospital samples, beta-lactam resistance genes in 8 samples, and tetracycline resistance genes in all 10 samples.

#### 3.4.2. Risks Associated with Separation

When food waste is source-separated from other forms of waste, it tends to contain fewer contaminants than when food is mixed with other materials and separated during recovery [56,106]. When food is co-mingled with other municipal solid waste (MSW), it encounters contaminants that are difficult to predict and quantify due to the “diversity in range and in scale” of chemicals [75] and other hazardous materials in mixed waste streams, due in part to the “poor availability of recycling facilities for hazardous wastes” [56]. For example, source-separated household wastes typically contain smaller amounts of heavy metals than those that were mechanically separated after collection [107,108,109]. While programs that require individuals to source-separate food waste are likely to result in less contamination [56,106], single-stream programs are often favored based on economic and social considerations due to higher levels of participation and thus recovery rates and lower transportation and collection costs [110]. Here, we see a prime example of how some stakeholders might prioritize environmental risk reduction while others prioritize reducing the risk that high transportation or collection costs make the system economically unsustainable, or that inconvenience might reduce access and participation and thus ultimately compromise environmental benefits. Many of these tradeoffs require better data so that decision-makers can model the potential impacts of waste alternatives.

#### 3.4.3. Risks Associated with Collection

Some studies suggest that opt-in services like subscription-based curbside collection and drop-off programs tend to have less contamination because participants elect to take part and are intrinsically motivated [76]. According to this logic, universal curbside collection programs or food waste disposal bans may introduce more contaminants because participants are less familiar with requirements and have less interest in the program. Stakeholders noted that participants in mandatory programs might be untrained, uninterested, unaware, or even resentful of their obligation to participate [50]. Research testing contamination in food waste samples from three New England states, however, failed to generate support for these assumptions. A recent study conducted by members of our team found that non-grocer samples from Maine where there is no mandate for recycling food waste contained similar levels of contamination to samples from regulated states [74]. This is perhaps due to large investments in education and outreach in the regulated states including, for example, Vermont’s “Let’s Scrap Food Waste” program that included comprehensive guidance and outreach for municipalities, businesses, institutions, haulers, and households [66].

There are also potential social risks associated with the regulatory environment for food waste recycling. Voluntary programs come with a risk of low participation rates. This is particularly true for subscription-based programs that can limit accessibility to wealthier households and institutions. Mandatory programs can ensure higher participation rates, particularly if publicly supported, to ensure widespread and fair access to recycling services. Some studies suggest that mandatory collection can also have significant social benefits. Reports from Vermont, for example, suggest that the implementation of their universal recycling law was accompanied by a 40% increase in food donations to hunger relief organizations as farmers, food processors, grocers, and caterers chose to donate food rather than send still edible foods for nutrient recovery [66]. There may also be some economic benefits for municipalities who are likely to see their landfill tipping fees significantly reduced.

Households generate large quantities of wasted food [1], and may participate in a range of collection regimes, ranging from food scrap drop-off programs to paid curbside collection subscription services or municipal curbside collection programs. Factors like the size and mobility of the collection containers, frequency of pick up, or the hours of operation at collection facilities can affect participation rates and contamination risk [111]. Karim Ghani and colleagues’ [112] study of household intentions to participate in backyard composting found that participants, in general, preferred the convenience of curbside pickup. Two pilot studies in Southern Maine found significant increases in participation and recycling rates when curbside collection was offered free of charge, but some studies found that plastic bag contamination was an issue as households used bags to contain what they refer to as the “ick” factor [50].

#### 3.4.4. Risks Associated with Processing Technology

There are many emerging strategies to recover value from wasted food, including mechanical biological treatment/biorefining and advanced thermal processing, among others [110]. Here, we focus on anaerobic/co-digestion and composting (see Table 2), due to the prevalence of these processes and strong growth in policy support. Importantly, anaerobic/co-digestion and composting have a role in circular food systems: both processes produce residuals that can be applied in agricultural production. Composting and digestion systems have similar and unique exposure to emergent risks. These risks range from visible contaminants to invisible process inhibitors and are shaped in part by the source of food waste and, in turn, affect the quality and reliability of the output available to end-users.

## 4. Discussion: Social, Economic, and Environmental Tradeoffs

To meet organics diversion goals, businesses, institutions, and municipalities are adopting a wide variety of strategies for food waste recycling, which are supported by regulatory agencies [57]. However, variable waste streams and recovery processes yield materials that are highly uneven in quality and energy content. Points of waste generation (institutional vs. residential generators), collection processes (source-separated vs. commingled), training processes (for people sorting and handling wastes), and treatment options (composting vs. digestion) all affect the potential quality, cost of management, social acceptability of outputs, and safety of the material.

Although our analysis focuses on generation, separation, collection, and processing, it is critical to note that to build a circular food system, residuals from composting and digestion processes must be applied *back to agricultural soil*. In order to make sure that happens, soil amendments must be safe and end-users must trust that they will improve soil nutrition and health. The perceived risks of end-users are informed by both the generators of food waste as well as the processing techniques used to create the residual material. Critically, these perceived risks may not closely track with measured risks [113] or the perceived risks of other participants in the circular food system. For example, while our stakeholders were concerned with contamination from herbicides and pesticides, Rahmani and colleagues found that compost users in Florida were most concerned about the presence of weed seeds in finished compost, while “adverse reaction to herbicide or pesticide residues was the least important barrier from the compost users point of view” [114]. Understanding the gaps between perceived and measured risks, then, is critical to building more circular food systems.

Further, because food waste is a complex problem, it is difficult for stakeholders—or indeed, researchers—to understand the ways in which unseen or unanticipated factors might disrupt food recovery and recycling systems. Economic considerations tend to be first and foremost among concerns for municipalities and businesses as they investigate participating in the circularization of the food system. Research in the social sciences has demonstrated that individuals base their perceptions of risk on heuristics—cognitive shortcuts that allow individuals to make decisions in the face of uncertainty or complexity [115]—further rationalizing research that helps to make emergent risks more explicit.

We see this information as essential as New England aspires to and continues to invest in more circular and localized food systems. The safety of the soil amendments produced from food waste is crucial for public health and necessary to maintain trust in food waste recovery processes as well as the resulting agricultural supplements. Research suggests that perceptions of risk and trust in waste management practices can significantly influence markets for agricultural supplements produced from waste streams [116,117,118].

New regulations and organics recovery targets produce new market opportunities for businesses seeking to recover soil amendments and/or energy from discarded organic materials. The value and social acceptance of the use of processed residuals will depend on both (1) the quality of the product, which is related to input material quality as well as processing of the discarded materials, and (2) the end-users’ trust in the product. It may be that it will take considerable time and educational effort to transform surplus food from waste to resources. As Riding and colleagues argue, “This can only be achieved by well-informed interactions between scientists, regulators and end users, to improve the spread and speed of innovation with this sector” [118].

## 5. Conclusions

Our contribution toward efforts to create safe and sustainable circular food systems, here, has been two-fold. First, we outlined a wide range of the social, economic, and environmental risks that food waste processors face. Some of these risks are well known to processors, while others are not and thus are often overlooked in risk mitigation planning. Similarly, while researchers are often aware of environmental risks, we find that many academic papers about food waste recycling fail to consider the economic and social risks that waste managers must also consider. Our hope is that by synthesizing the potential risks we see in the literature and in stakeholder perspectives, we might inform policy formation as governments, on multiple scales, attempt to reduce food waste and ensure that wasted food nutrients are cycled back into food production. The identification of potential risks can help to stimulate progress toward risk mitigation in organics management policy. Secondly, this work also synthesized research from multiple disciplines and stakeholder perspectives to create a conceptual model of potential contamination risks as mediated by differences in waste generation, sorting, collection, and processing. We hope this contribution will draw attention to the interconnectedness of food waste systems such that decision-makers can envision how economic, environmental, and social risks can be amplified or attenuated in a circular food system. We argue that our stakeholder-informed and transdisciplinary approach is important given the complex nature of food systems located in interconnected economic, social, and environmental systems. The geographical and topical limitations of our study could be remedied by future research, which would broaden the analysis of potential risks in other geographies, across the scale and for food waste solutions beyond food waste nutrient cycling.

## Figures and Tables

**Table 1:** Potential Economic, Social, and Environmental Risks to be Mitigated.

Type of Risk	Description
Section 3.1 Economic	
*Section 3.1.1 Investment risks*	Concerns about whether investments in food waste nutrient cycling will yield adequate returns.
*Section 3.1.2 Market risks*	Risks associated with the supply of food waste inputs (participation) and demand for finished products (market strength/consumer trust).
Section 3.2 Social	
*Section 3.2.1 Subverted incentives with social impacts*	Risks associated with undermining the Wasted Food Scale (e.g., diverting attention and investment in reduction and redistribution).
*Section 3.2.2 Environmental injustice*	Risks associated with an uneven distribution of the costs and benefits of more circular food systems.
Section 3.3 Environmental	
*Section 3.3.1 Physical contaminants*	Physical objects: microplastics, glass, and trash that enter composting and AD systems, and have the potential to disrupt agricultural systems.
*Section 3.3.2 Trace contaminants*	Unseen environmental risks to food waste recycling.
*Section 3.3.2a Biological agents*	Pathogens and antibiotic resistance genes can cause harm to processors as well as end-users of soil amendments.
*Section 3.3.2b Process inhibitors*	Materials that have negative impacts on the operation of composting and digestion processes.
*Section 3.3.2c Heavy metals*	Toxic trace metals (e.g., Zn, Cu, Cd) may be taken up from the soil, or contaminate food products from packaging materials or during processing or handling.
*Section 3.3.2d Toxicants*	Pesticides, herbicides, and other toxic organics (e.g., PFAS) that can pose risks for soil amendments produced from wasted food.

**Table 2:** Comparison of processing methods for MSW organics.

Method	Process	Applicability	Risks
Compost	Relatively simple to set up and operate Able to accept a variety of material and adaptable to changes in input materials Produces useful soil amendment Can, in some cases, recover heat energy Scale can vary (100s—10,000+ tons per year)	Input composition is flexible, but length of treatment and need for bulking agents may be affected Relatively high volume of residual to be land applied—requiring a large market Low start-up costs Large land area required for processing and storage of bulking agents and final product Lower or no water input required	Volatile components may be transferred to the air Some organics may not biodegrade and remain in the residual applied to soil Most metals and plastics will remain in the residual Economically difficult at a small scale and difficult to secure necessary volume for large-scale rural operations Odor, pest, and traffic concerns
Anaerobic Digestion	More complex operation than composting requiring skilled operators, but mature technology More highly affected by input material composition and mixing Long start-up and recovery times Produces compressible fuel or heat and electricity and useful soil amendment	Best applied when input material is consistent over time to avoid system disruption and poor performance Some materials may not be degraded under anaerobic conditions (or require additional pre-hydrolysis)	Long recovery time required after system failure due to high ammonia, salts, metals, or other process inhibitors Some organics not degraded. Metals and recalcitrant organics will end up in liquid and solid fractions High fixed costs Methane is explosive

## Data Availability

All data generated via stakeholder working groups, surveys, and interviews are archived with the Senator George J Mitchell Center for Sustainability Solutions. De-identified data are publicly available upon request in compliance with the University of Maine’s Internal Review Board protocols for research with human subjects.
